# Pharmacokinetic and pharmacodynamic modeling of cyadox against *Clostridium perfringens* in swine

**DOI:** 10.1038/s41598-017-03970-9

**Published:** 2017-06-22

**Authors:** Lei Yan, Shuyu Xie, Dongmei Chen, Yuanhu Pan, Yanfei Tao, Wei Qu, ZhenLi Liu, Zonghui Yuan, Lingli Huang

**Affiliations:** 1 National Reference Laboratory of Veterinary Drug Residues (HZAU) and MOA Key Laboratory for Detection of Veterinary Drug Residues, Huazhong Agricultural University, Wuhan, Hubei 430070 China; 2 MOA Laboratory for Risk Assessment of Quality and Safety of Livestock and Poultry Products, Huazhong Agricultural University, Wuhan, Hubei 430070 China; 3 Hubei Collaborative Innovation Center for Animal Nutrition and Feed Safety, Huazhong Agricultural University, Wuhan, Hubei 430070 China

## Abstract

The purpose of this study was to evaluate the activity of cyadox against *Clostridium perfringens* in swine and optimize the dosage regimen using *ex vivo* pharmacokinetic-pharmacodynamic (PK-PD) modeling. After oral administration, the ileum fluid of pigs containing the free cyadox was collected by implanted ultrafiltration probes. The T_max_, AUC_24h_, and CL/F of free cyadox in the ileum fluid were 1.96 h, 106.40 μg/h/mL, and 0.27 L/kg/h, respectively. Cyadox displayed a concentration-dependent killing action against *C*. *perfrignens*. The minimum inhibitory concentration (MIC) of cyadox against 60 clinical isolates ranged from 0.5 to 8 μg/mL, with MIC_50_ and MIC_90_ values of 2 and 4 μg/mL, respectively. The MIC was 2 μg/mL against the pathogenic *C*. *perfrignens* isolate CPFK122995 in both broth and ileum fluid. According to the inhibitory sigmoid E_max_ modeling, the AUC_24h_/MIC ratios of ileum fluid required to achieve the bacteriostatic, bactericidal, and virtual bacterial elimination effects were 26.72, 39.54, and 50.69 h, respectively. Monte Carlo simulations for the 90% target attainment rate (TAR) predicted daily doses of 29.30, 42.56, and 54.50 mg/kg over 24 h to achieve bacteriostatic, bactericidal, and elimination actions, respectively. The results of this study suggest that cyadox is a promising antibacterial agent for the treatment of *C*. *perfringens* infections, and can be used to inform its clinical use.

## Introduction

*Clostridium perfringens* is a serious enteric pathogen affecting pigs, especially the toxigenic types A and C^1–4^. *Clostridium perfringens*-associated enteropathy is a highly lethal disease of the digestive system characterized by diarrhea and hemorrhagic and necrotic enteritis in piglets, associated with a high mortality rate, decreased daily weight gain, increased feed conversion ratio, and devastating economic losses for the livestock industry^1, 5^. Moreover, the high infection rate of *C*. *perfringens* in pigs, combined with the risk of transmission to the human food chain, represents a public health hazard^6, 7^. Increasing attention has been focused on the control of this disease. Unfortunately, conventional drugs achieve unsatisfactory clinical efficacy because the bacteria readily develop widespread resistance^8–11^. The incidence of resistance for tetracycline and minocycline against *C*. *perfringens* isolated from different animal sources has been reported to be 33–73% and 17–40%, respectively^12^. A study by Rood *et al*. that evaluated 258 strains isolated from pigs provided antibiotic-containing feed found that 200 strains were resistant to tetracycline and 58 strains were simultaneously resistant to erythromycin, clindamycin, and lincomycin^9^. According to a report by Slavic, 28% and 31% of pig-derived strains isolated in Ontario exhibited reduced susceptibility to clindamycin and erythromycin, respectively^8^. Therefore, it is necessary to find novel alternative antimicrobial compounds in order to combat the increasing emergence of resistance to available antibiotics.

**Figure 1:**
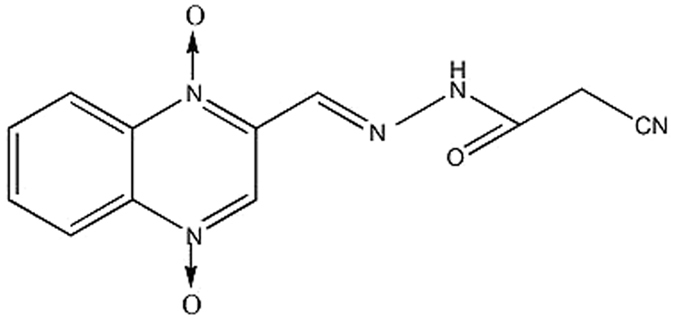
Chemical structure of cyadox (CAS No.:65884-46-0, MW = 271).

Cyadox (Fig. 1) is a derivative of the synthetic quinoxaline-1,4-di-N-oxides (QdNOs), which have been used in animal production as a potent antimicrobial agent against most pathogenic bacteria since it was first introduced in the 1970s^13–15^. Previous studies have demonstrated that cyadox possesses excellent antibacterial activity against enteric pathogens of animal origin such as *Salmonella* and *Escherichia coli* ^16^. Cyadox is hypoxia-selective drug that exhibits much stronger activity in the absence of oxygen^17, 18^. Therefore, cyadox may be active against *C*. *perfringens*, an obligate anaerobe pathogen. Importantly, when compared with its well-known congeners carbadox and olaquindox, cyadox shows much lower toxicity and higher safety according to systematic toxicological and microbiological safety evaluations^19–23^. Cyadox is expected to have potential wide applications for controlling enteric disease caused by *C*. *perfringens*.

When developing and evaluating new antimicrobial drugs, optimizing the dosage schedule is essential to achieve clinical cures, in addition to minimizing the emergence of antimicrobial drug resistance. Pharmacokinetic-pharmacodynamic (PK-PD) modeling reflects the relationship between the drug, bacteria, and the animal affected, as well as quantifying the potency and efficacy of antimicrobials against target pathogens. Pharmacokinetic-pharmacodynamic analysis has become an important tool for determining the optimal dosage regimen^24, 25^. Furthermore, both the US Food and Drug Administration (FDA) and European Medicine Agency have recommended assessing the PK/PD relationship for new antimicrobial compounds^25^.

It is generally recognized that the concentration of the free form of an antibiotic at the infection site is responsible for its antibacterial effect^26^, especially for local infection. Due to the difficulty in determining the free drug concentration at the target site, the PK data in most previous PK-PD studies have often been obtained from plasma. It has been shown that the antibiotic concentration time-course in plasma is significantly different from that in biophase at the infection site, including the interstitial fluid (ISF) and epithelial lining fluid (ELF)^27–29^. Therefore, it is necessary to determine the active concentration of free antimicrobial drugs at the target site using PK-PD modeling to achieve a rational dosage regimen. Previous studies have reported the application of an ultrafiltration sampling technique for isolating the free (unbound) drug in the gastrointestinal tract of calves, and in the ISF of different tissues (e.g., intramuscular, subcutaneous, and intrapleural tissues) of calves, sheep, and pigs^27, 28, 30, 31^.

In this study, the antibacterial activity of cyadox against pathogenic *C*. *perfringens* of pig origin was investigated, and the recommended daily dose was calculated based on PK-PD modeling following the collection of free cyadox in the small intestine by ileum ultrafiltration probes. The aim of this study was to evaluate the effectiveness of cyadox for the treatment of *C*. *perfringens*-associated enteropathy in order to inform its clinic use.

## Results

### Minimum inhibitory concentration distribution of cyadox against clinical stains of *Clostridium perfringens*

**Figure 2:**
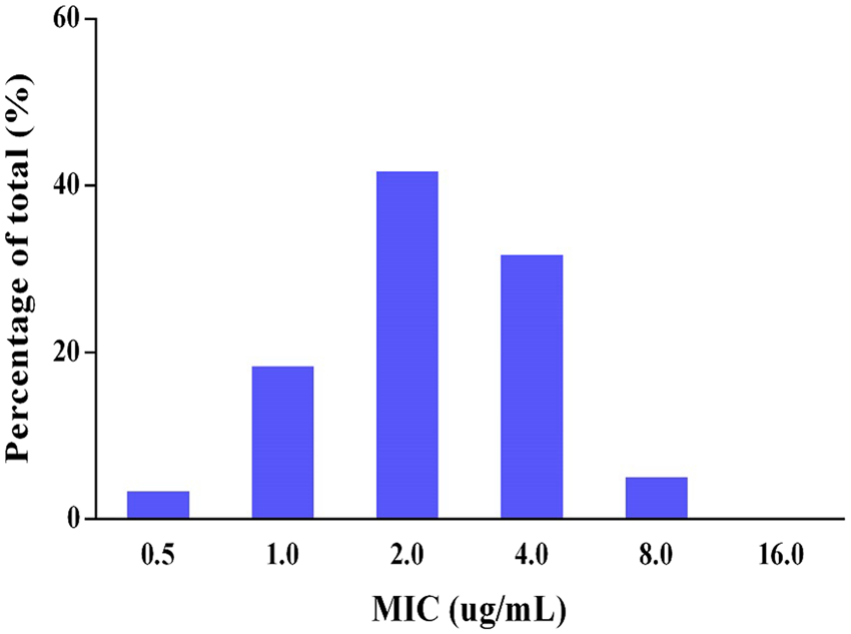
MIC distribution of cyadox against pig origin clinical *Clostridium perfringens* (60 strains).

The minimum inhibitory concentration (MIC) distribution of cyadox against the sixty clinical strains of *C*. *perfringens* is shown in Fig. 2. The MIC values ranged from 0.5 to 8 μg/mL. The corresponding MIC_50_ and MIC_90_ were 2 and 4 μg/mL, respectively, suggesting that cyadox displays a potent antibacterial effect against anaerobic *C*. *perfringens*.

### Minimum inhibitory concentration, minimum bactericidal concentration, and post-antibiotic effect of cyadox against CPFK122995

The pathogenic CPFK122995 strain with an MIC close to the MIC_50_ was chosen as the representative strain to investigate the antibacterial activity of cyadox *in vitro and ex vivo*. The MIC and minimum bactericidal concentration (MBC) values of cyadox against *C*. *perfringens* CPFK122995 were 2 and 4 μg/mL in Brucella broth and 2 and 8 μg/mL in pig ileum fluid, respectively. The MBC/MIC ratios were 2:1 and 4:1, respectively, suggesting a relatively gentle concentration-effect relationship of cyadox. The mutant prevention concentration (MPC) of cyadox against CPFK 122995 was 12 μg/mL. The mean post-antibiotic effect (PAE) values following exposure to different concentrations of cyadox for 1 and 2 h are shown in Table 1.

**Table 1:** The PAE of cyadox against *Clostridium perfringens* CPFK 122995.

Drug concentration (μg/mL)	PAE (h)	
	Exposure of 1 h	Exposure of 2 h
2 (1 × MIC)	0.85	1.26
4 (2 × MIC)	0.99	1.87
8 (4 × MIC)	1.02	2.35

### *In vitro* and *ex vivo* antimicrobial activity

**Figure 3:**
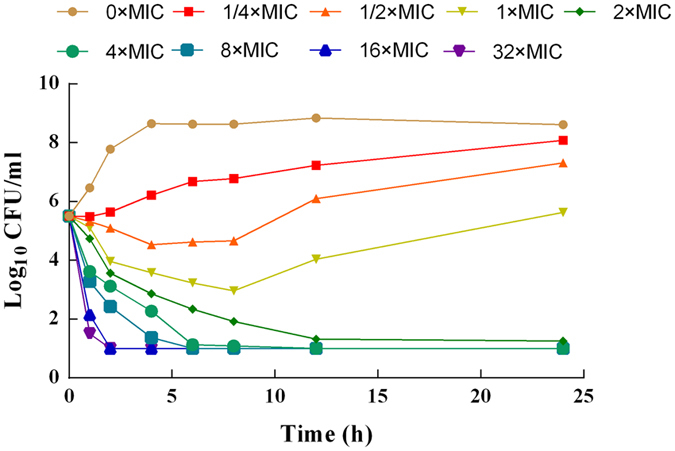
*In vitro* killing curves of cyadox against *C*. *perfringens* CPFK 122995 in brucella broth (mean, n = 3). SEM bars not shown for clarity.

The *in vitro* time-kill curves of cyadox against *C*. *perfringens* CPFK122995 are illustrated in Fig. 3. According to the profiles, cyadox displayed a concentration-dependent bactericidal effect as increasing drug concentrations induced more rapid and radical killing effects. Persisting inhibition of bacterial growth was observed only within the first 6 h for the Brucella broth containing a drug concentration lower than 2 × MIC. When *C*. *perfringens* was exposed to cyadox at a concentration equal to or higher than 2 × MIC, the bacteria were significantly decreased to the undetectable level (≤10 CFU/mL) after 24 h of incubation.

**Figure 4:**
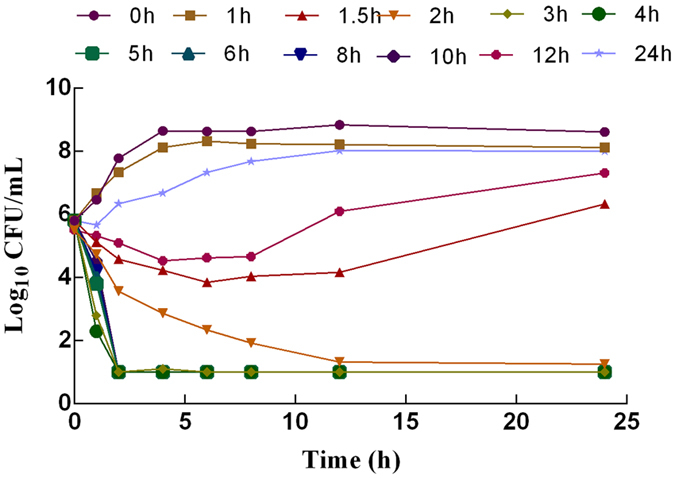
*Ex vivo* killing curves of cyadox in ileum fluid against *C*. *perfringens* CPFK122995 (mean, n = 3). SEM bars not shown for clarity.

The *ex vivo* kill curves of cyadox against *C*. *perfringens* CPFK122995 in the ileum fluid of pigs after oral administration are shown in Fig. 4. The bacteria were drastically reduced to the undetectable limit (<10 CFU/mL) after exposure to the ileum fluid collected between 1.5–8 h after oral administration, suggesting that cyadox exhibited a concentration-dependent killing mechanism in the *ex vivo* environment, consistent with the *in vitro* bactericidal effect. Slight regrowth of bacteria exposed to the ileum fluid samples collected at 1.5 and 12 h after oral administration was observed after incubation for 6 h.

### Pharmacokinetics of cyadox in the plasma and intestinal tract

**Figure 5:**
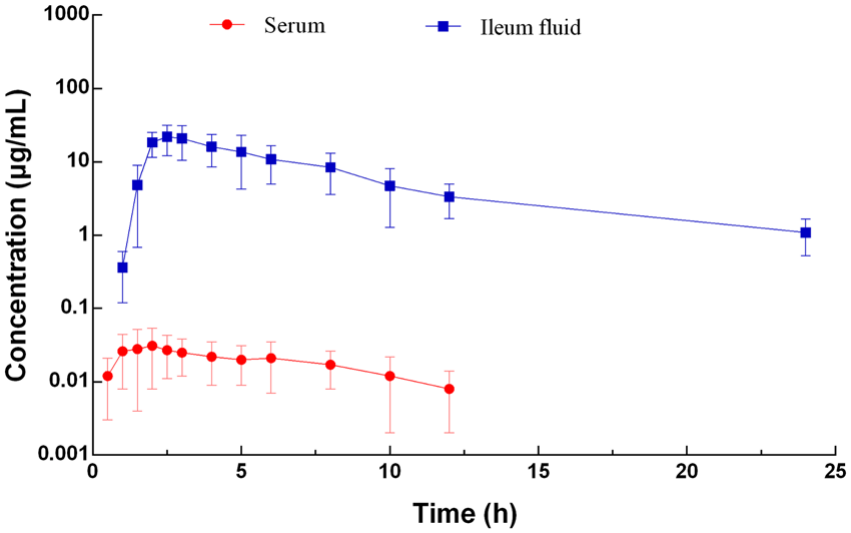
Concentration vs. time curve of cyadox in plasma and ileum fluid of pigs (Mean ± SD, n = 6).

The mean concentrations of cyadox in the plasma and ileum fluid after oral administration at a dose of 30 mg/kg are presented in Fig. 5. The plasma drug concentrations were found to contain both free and protein-bound cyadox, whereas only the microbiologically active free drug was found in the ileum fluid collected using the ultrafiltration probe. The absorption of cyadox after oral administration was limited, reaching the maximum plasma concentration (C_max_ = 0.031 μg/mL) at 2.41 h. After this point, the concentration of the drug quickly decreased to the quantification limit (0.02 μg/mL) by 12 h. The pharmacokinetic parameters derived from non-compartmental analysis are presented in Table 2, and the area under the concentration-time curve (AUC)_0–24h_ of plasma was 0.22 μg/h/mL. Compared to the cyadox concentration in plasma, the concentration in the ileum fluid was markedly higher, with a C_max_ of 23.66 μg/mL at 1.96 h, and the AUC_0–24h_ (106.40 μg/h/mL) was also much higher. The distribution and elimination of cyadox in the ileum fluid were rapid, with a T_1/2λ_ of 5.86 h (Table 2).

**Table 2:** Pharmacokinetic parameters of cyadox in plasma and ileum fluid (mean ± SD, n = 6).

Variables (units)	Plasma	Ileum fluid
C_max_ (μg/mL)	0.03 ± 0.03	23.66 ± 0.92
T_max_ (h)	2.41 ± 1.32	1.96 ± 0.03
T_1/2 λz_ (h)	3.61 ± 1.87	5.86 ± 0.71
AUC_0–last_ (μg ∙ h/mL)	0.22 ± 0.16	106.40 ± 12.68
AUC_0–∞_ (μg ∙ h/mL)	0.26 ± 0.21	112.35 ± 14.07
AUMC_0–last_ (μg ∙ h^2^/mL)	1.14 ± 0.42	568.49 ± 110.27
AUMC_0–∞_(μg ∙ h^2^/mL)	1.86 ± 0.92	743.23 ± 165.71
MRT_0–last_ (h)	5.19 ± 3.21	5.34 ± 0.39

Notes: The pharmacokinetic parameters were calculated by non-compartment model.

### Pharmacokinetic-pharmacodynamic integration and modeling

The PK-PD parameters obtained from integration of the *in vivo* PK data with the *in vitro* MIC and MPC values are shown in Table 3. The *ex vivo* AUC_0–24h_/MIC and AUC_0–24h_/MPC ratios of cyadox against *C*. *perfringens* CPFK122995 were 66.39 and 11.07 h, respectively. The times required for the drug concentration in the ileum fluid to become higher than the MIC (T > MIC) and MPC (T > MPC) were 10.92 and 3.65 h, respectively. The mean C_max_/MIC and C_max_/MPC ratios of cyadox in the ileum fluid were 10.79 and 5.40, respectively.

**Table 3:** PK-PD integration of cyadox in pig ileum fluid agianst *Clostridium perfringens* after oral administration at a dose of 30 mg/kg (mean ± SD, n = 6).

Parameters	Units	Mean ± SD
C_max_/MIC	—	10.79 ± 0.82
AUC_0–24h_/MIC	h	66.39 ± 12.47
T > MIC	h	10.92 ± 2.54
C_max_/MPC	h	1.80 ± 0.25
AUC_0–24h_/MPC	h	11.07 ± 1.53
T > MPC	h	3.65 ± 0.83

**Figure 6:**
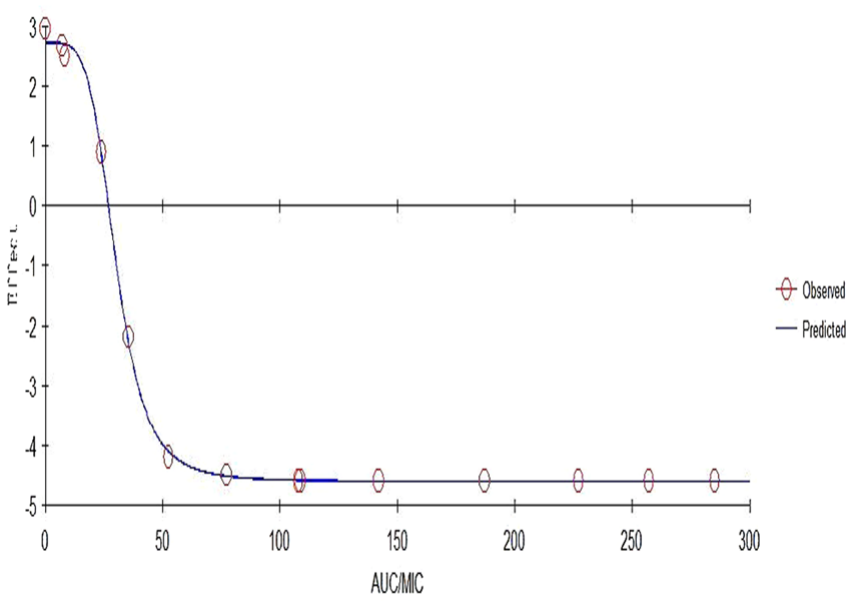
Plots of *ex vivo* AUC/MIC ratios versus the amount difference of *C*. *perfringens* CPFK122995 between 0 and 24 h in *ex vivo*.

The relationship between the antimicrobial efficacy and the *ex vivo* PK/PD parameter of AUC_0–24h_/MIC ratios was fitted by using the inhibitory sigmoid E_max_ model. The model parameters, including the Hill coefficient (N), E_0_, E_max_, and AUC_0–24h_/MIC values for the three levels of growth inhibition are presented in Table 4. As shown in Fig. 6 and Table 4, the AUC_0–24h_/MIC values for bacteriostatic activity (E = 0), bactericidal activity (E = −3), and a virtual elimination effect (E = −4) were 26.72, 39.54, and 50.69 h, respectively.

**Table 4:** PK-PD modeling of *ex vivo* cyadox after oral administration at a dose of 30 mg/kg.

Parameters	Units	Values
E_max_	Log CFU/mL	2.71 ± 0.37
E_0_	Log CFU/mL	−4.61 ± 0.69
E_max_−E_0_	Log CFU/mL	7.32 ± 1.06
EC_50_	h	30.07 ± 6.94
N (slope)	/	4.62 ± 0.72
AUC_24h_/MIC for bacteriostatic effect	h	26.72 ± 6.28
AUC_24h_/MIC for bactericidal effect	h	39.54 ± 7.31
AUC_24h_/MIC for eradication effect	h	50.69 ± 9.16

### Predicted daily dosage

The predicted daily doses based on the distribution of the CL/F and AUC_0–24h_/MIC ratios for the three levels of antibacterial effects derived from PK/PD modeling and MIC distributions are presented in Table 5. The doses predicted to obtain bacteriostatic, bactericidal, and elimination effects for *C*. *perfringens* over 24 h were 13.2, 19.70, and 25.46 mg/kg for the 50% target attainment rate (TAR), and 29.30, 42.56, and 54.50 mg/kg for 90% TAR.

**Table 5:** Predicted daily dosages based on PK-PD modelling.

Predicted daily doses	Target attainment rate	
	50%	90%
Bacteriostatic (E = 0)	13.2	29.3
Bactericidal (E = −3)	19.70	42.56
4log10 reduction in count (E = −4)	25.46	54.50

## Discussion

*Clostridium perfringens* is an anaerobic, spore-forming bacterium that produces a variety of toxins, responsible for a wide range of diseases in humans and animals for food purposes^32^. Moreover, the control of *C*. *perfringens* is very difficult due to the rapid growth and resistance of pathogenic bacteria to commonly used antimicrobial drugs, resulting from overuse and misuse^33^. Therefore, new antimicrobial agents, particularly those without cross-resistance to existing drugs, are required to effectively manage necrotic enteritis. Cyadox, a candidate of the important QdNOs, represents a promising antibacterial agent for the treatment of necrotic enteritis. This is due to the demonstrated hypersensitivity of clinical *C*. *perfringens* strains to this antibiotic combined with the reduced likelihood of cross-resistance to commonly used antimicrobial drugs, as cyadox works via a different antibacterial mechanism. In this study, the MIC_50_ and MIC_90_ of cyadox were found to be 2 and 4 μg/mL, respectively, suggesting that cyadox possesses a satisfactory potency against the 60 isolates tested. Moreover, non-bimodal distributions with low MIC values were observed (Fig. 2), indicating that resistance of the tested isolates did not emerge. Based on the results of the susceptibility test, cyadox is expected to be an ideal drug for the treatment of necrotic enteritis in swine. In order to determine the rational dosage regimen, the *ex vivo* PK/PD relationship of cyadox against *C*. *perfringens* in the small intestinal fluid of pigs was evaluated.

Firstly, the highly pathogenic clinical isolate CPFK122995 was selected in accordance with a pathogenicity experiment conducted on mice (data not shown) in order to study the *in vitro* and *ex vivo* pharmacodynamics. The MICs obtained for the Brucella broth and ileum fluid were not significantly different, indicating that the composition of the growth matrix does not affect the antibacterial activity of cyadox. The kill curve and PAE showed that cyadox has bactericidal activity against *C*. *perfringens*, demonstrating that this antibiotic is concentration-dependent and has a certain PAE (0.85–2.35 h). Against CPFK122995, cyadox resulted in a >4log_10_ reduction in viable bacterial count after 24 h of exposure, with the viable counts typically reduced to lower than the LOD of the assay. As cyadox was found to be a concentration-dependent antibiotic, the *ex vivo* AUC/MIC should be selected for PK-PD modeling, according to the reports. The regrowth phenomenon of bacteria after incubation for 6 h occurred *in vitro* killing curves when the concentration below the MBC and in *ex vivo* killing curves when they exposed to the ileum fluid sample collected at 1.5 and 12 h after oral administration might be due to the growth vigor recovery of suppressed bacterial as the degradation of antibiotic during incubation. Elisabet *et al*. confirmed that the degradation of antibiotic was statistically significant during incubation in time-kill curve experiments^34^.

Secondly, the PK of cyadox at the target site (ileum fluid) was studied in healthy pigs. Furthermore, experts have emphasized that measurement of unbound biological drug concentrations, not total drug concentrations, is important for evaluating the antimicrobial activity^35^. Therefore, the active unbound drug concentrations were determined in the current study to provide a better correlation with the microbiological outcome from PK-PD modeling. *C*. *perfringens* types A and C are the principal enteric pathogens of swine and could induce disease when they are proliferated to 10^8^ to 10^9^ CFU/g in gastrointestinal contents under appropriate conditions^1^. This study investigated the PK of free cyadox in the gastrointestinal tract (GIT) of pigs for the first time, achieved via pre-implanted ultrafiltration probes. The *in vivo* ultrafiltration intestinal microsampling technique was found to be an effective method for collecting the free drug in the GIT of pigs following oral administration of cyadox. The probes had no postoperative complications, were well tolerated by pigs, and provided the ability to obtain an adequate number of sequential protein-free ileum fluid samples without having to sacrifice animals or risk leakage, dislodgement, and peritonitis^36^. More importantly, the *in vivo* ultrafiltration probes allowed the target animals to maintain a normal physiological state, which is advantageous as a more accurate PK study can be achieved^37^.

In the PK study, the C_max_ and AUC_24h_ of cyadox in plasma were found to be 0.031 μg/mL and 0.22 μg/h/mL after oral administration at a dose of 30 mg/kg. These results are consistent with Zhao, who reported that the C_max_ and AUC_24h_ of cyadox in plasma were 0.043 μg/mL and 0.38 μg/h/mL, respectively, after an oral dose of 40 mg/kg^38^. Compared to plasma, the drug concentrations in the ileum fluid were significantly higher with C_max_ and AUC_24h_ values of 23.66 μg/mL and 106.40 μg/h/mL, respectively. The large difference in cyadox concentrations between these sample types may be due to the high amount of biliary excretion or limited absorption after oral administration. The high drug concentrations in the ileum indicated that cyadox could have a favorable antibacterial effect in the GIT after oral administration^39^. Furthermore, the values for terminal elimination half-life (T_1/2 λz_) and T_max_ in the ileum fluid were 5.86 and 1.96 h, respectively. According to the PK in the ileum fluid, cyadox appears to be suitable for the treatment of GIT in pigs (e.g., necrotic enteritis).

Pharmacokinetic-pharmacodynamic integration and modeling, which could represent a better approach compared to dose titration studies for formulating rational dosage regimens in veterinary medicine, was established to determine the rational dosage regimen of cyadox for necrotic enteritis therapy.

For the PK/PD integration process, the PK parameters for free cyadox in the ileum fluid were integrated with the MIC data (*in vitro* and *ex vivo*) using CPFK122995 as a typical pathogenic strain of *C*. *perfringens*. The PD data from the ileum fluid were used to predict clinically relevant dosage regimens as the ileum fluid is a more clinically relevant matrix than broth. The ileum fluid samples were collected by the implanted ultrafiltration devices since the *C*. *perfringens* types A often resides in the jejunum and ileum of pig^1^. The cannulation collection devices were usually used to collect the ileum contents of swine for the measurements of the effects of antibiotics on intestinal bacteria^36^. For example, Wang *et al*. use this device to study on PK/PD modeling of enrofloxacin against *E*. *Coli* in swine^40^. Unfortunately, the method could not effectively separate the total and free drug. In recently, Foster *et al*. found that the implanted ultrafiltration devices was a useful tool to collect the free drug in ileum and colon fluid of calves for measuring the antibacterial effect on enteric bacteria *Enterococcus faecalis* or *Salmonella entarica* ^27^. The values for Cmax/MIC, AUC/MIC, and T > MIC were 10.79, 66.39, and 3.65 h, respectively. In terms of the antibacterial properties of cyadox against *C*. *perfringens*, the inhibitory sigmoidal E_max_ model was used to model the PK/PD. The dose-response profile of cyadox showed a significant correlation between the observed and predicted profile with the *ex vivo* antibacterial efficacy against *C*. *perfringens* (R^2^ = 0.999). Based on the equation, the *ex vivo* AUC_0–24h_/MIC ratios required to achieve the three levels (bacteriostatic, bactericidal, and virtual elimination effect) of antibacterial activity in the ileum fluid of pigs were 26.72, 39.54, and 50.69 h, respectively. The *in vivo* AUC_0–24h_/MIC ratio (66.39 h) was higher than the *ex vivo* AUC_0–24h_/MIC ratio required for the bactericidal effect against the *C*. *perfringens* CPFK122995 isolate, with a MIC value of 2 μg/mL. This suggests that the administered daily dose of 30 mg/kg body weight could guarantee clinical efficacy against infections associated with *C*. *perfringens*, with a MIC_50_ value of 2 μg/mL. According to the dosage calculation equation, the central tendency and measure of dispersion of the PK parameters and the MICs against clinic isolates are required to describe the possible range of clinical dosages. Based on the Monte Carlo simulations, the predicted daily dose for 50% TAR and 90% TAR to provide a 3log^10^ reduction in bacterial count were found to be 19.70 and 42.56 mg/kg, respectively. However, it should be noted that the bacterial endpoint under *in vivo* conditions may differ from the predicted dose based on *ex vivo* data, as the host’s immune system is likely to be an important factor contributing to bacterial eradication^37^. For the Monte Carlo simulation, it is necessary to obtain PK parameters from a large population of animals. The small sample size used to calculate the PK parameters in this study might represent a limitation in terms of the conclusions that can be drawn from the simulations.

## Conclusions

The high concentration of cyadox found in the intestinal tract of pigs, combined with the high susceptibility of clinical *C*. *perfringens* isolates, suggests that cyadox is a promising drug for the treatment of *C*. *perfringens* infections. The unbound drug present in the intestinal fluid was collected by pre-implanted ultrafiltration probes, then used to simulate the rational clinic dosage using the PK-PD modeling in order to obtain a more effective dosage. The doses predicted to achieve bacteriostatic, bactericidal, and elimination effects against *C*. *perfringens* over 24 h were 13.2, 17.90, and 25.46 mg/kg for 50% TAR, and 29.30, 42.56, and 54.50 mg/kg for 90% TAR. The calculated recommended dose could assist in achieving more precise administration, increasing the effectiveness of treatment for *C*. *perfringens* infections while also avoiding resistance emergence. However, the suggested dose regimens should be validated in clinical practice.

## Materials and Methods

### Chemicals and reagents

Cyadox (99.8%) was provided by the Institute of Veterinary Pharmaceuticals (HZAU, Wuhan, China). Dimethyl sulfoxide (DMSO) was obtained from Amresco. Sodium carboxymethyl cellulose (CMC) was purchased from Sigma-Aldrich Chemie B.V. (Zwijndrecht, The Netherlands). Acetonitrile, formic acid, ethyl acetate, methanol, and dichloromethane were obtained from Tedia (OH, USA). Water was prepared with a Milli-Q system (Millipore, Bedford, MA, USA). All chemicals used in this experiment were analytical grade or higher.

### Bacteria

Sixty strains of *C*. *perfringens* isolated from pig farms in the Guangdong province of China were provided by the Department of Veterinary Pharmacology at South China Agricultural University. *Bacterioides fragilis* ATCC 25285, purchased from American Type Culture Collection (ATCC), served as the quality control for MIC determination. All strains were stored at −80 °C until use. Before each experiment, all bacterial cultures were subcultured on Brucella blood agar and incubated at 37 °C for 18–24 h.

### Animals

Six healthy, castrated, crossbred (Duroc × Large White × Landrace) pigs (weighing 15–20 kg) were purchased from the Livestock and Poultry Breeding Center of Hubei Province (Wuhan, China). The pigs were kept separately in all-steel cages and provided *ad libitum* access to water and antibiotic-free feed for 1 week to acclimatize. The temperature and relative humidity of the housing environment were kept at 18–25 °C and 45–65%, respectively. The use of pigs and all experimental protocols in this study were approved by the Institutional Animal Care and Use Committee at Huazhong Agricultural University (approval number HZAUSW-2016-007). Medical measures such as anesthetics were used to alleviate animal suffering and guarantee animal welfare during the trial.

### Ultrafiltration probe surgical procedure

Surgical implantation of the ileum ultrafiltration probe was conducted according to Warren *et al*. by a practicing veterinarian^41^. Briefly, food and water were withheld from the pigs for 24 h prior to surgery. A14G venous indwelling needle was initially placed in the auricular vein of each pig. The animals were premedicated by intravenous administration of 0.1 mg/kg xylazine and flunixin. General anesthesia was induced by slowly injecting sodium pentobarbital saline via the intravenous indwelling needle. Once anesthetized, each pig was placed in the left lateral recumbency position on the operating table, then the right paralumbar fossa was clipped and scrubbed for sterile surgery. A vertical 10 cm skin incision was made approximately equidistant from the last rib to the tuber coxae in the paralumbar fossa. The collecting loops of an ultrafiltration probe (UF-3-12; BAS, West Lafayette, IN, USA) were then inserted into the lumen of the ileum, approximately 30 cm orad to the ileocecal orifice, and sutured into place, and the free ends of the probe were exteriorized cranial to the skin incision. Finally, the paralumber incision was closed in three layers. After the pigs had woken from anesthesia, the probes were prepared to collect fluid samples from the ileum of each animal.

### Animal study

Each pig was administered a single oral dose of cyadox (50 mL of a cyadox CMC suspension) at a dose of 30 mg/kg 48 h after surgery. The ileum fluid samples obtained using the probes placed in the ileum were collected by changing the vacutainer tubes, and blood samples were collected from the jugular catheter into heparinized tubes. Samples were collected 0, 0.5, 1, 1.5, 2, 2.5, 3, 4, 5, 6, 8, 12, and 24 h after drug administration. During the experimental period, pigs were allowed *ab libitum* water and antibiotic-free feed.

### Sample extraction

For the plasma extraction, 1 mL of the collected sample was mixed with 3 mL ethyl acetate and vortexed for 3 min. The supernatant was collected after centrifugation for 10 min at 8000 *g* and 4 °C, and the extraction was repeated. The supernatants obtained from the two extractions were mixed, then evaporated to dryness at 50 °C under nitrogen. The residue obtained after evaporation was reconstituted in 0.2 mL of mobile phase under vortex, filtered through a 0.22-μm membrane, then injected into a high-performance liquid chromatography (HPLC) instrument for detection.

For the ileum sample extraction, 2 mL of a solution containing metaphosphoric acid:methanol:water (1:50:49 v/v/v) was added to 0.2 mL of ileum fluid for cyadox extraction. The sample was extracted twice into sealed 10-mL tubes by firstly shaking for 10 min then centrifuging at 8000 *g* to collect the supernatant. The collected supernatant was back-extracted using 5 mL dichloromethane under vortex. After being left to stand for 3 min, the lower layer liquid was pooled and then dried under nitrogen at 50 °C. The residue was redissolved in 0.2 mL of mobile phase under vortex. The sample was injected into the HPLC detection vial after filtration through a 0.22-μm membrane.

### High-performance liquid chromatography method for cyadox determination

The cyadox present in the plasma and ileum fluid samples after extraction was analyzed using a Waters 2695 series HPLC and a Waters 2487 UV detector set at a wavelength of 306 nm, as described previously^42^. An Agilent ZORBAX SB-C_18_ column (250 × 4.6 mm i.d., 5 μm; Agilent Technologies, Santa Clara, CA, USA) was used for separation. The mobile phases were 0.1% formic acid and acetonitrile. The injection volume and flow rate were 20 μL and 1 mL/min, respectively.

The specificity of the cyadox detection method was good, and there was no endogenous interference on the chromatograms. The linear range for the standard curve of cyadox ranged from 0.02 to 0.5 μg/mL (r^2^ > 0.999) in plasma and 0.1 to 30 μg/mL (r^2^ > 0.999) in ileum fluid. The limits of quantification (LOQ) were 0.02 μg/mL in plasma and 0.1 μg/mL in ileum fluid. The mean recovery of cyadox was > 85% in the plasma and ileum samples. The coefficient of variability (CV%) was < 15% for both intra- and inter-day variation.

### Pharmacokinetic parameter analysis

The concentration-time data for cyadox in the ileum ultrafiltration fluid and plasma samples were analyzed for individual pigs by non-compartmental analysis (WIN-NONLIN; Pharsight Corporation, Mountain View, CA, USA) using the statistical moment approach^43^. The AUC of the intestinal concentration from 0 to 24 h were calculated by the linear trapezoidal method. The mean residence time (MRT) was determined as AUMC (area under the first moment curve)/AUC in non-compartmental analysis.

### Determination of minimum inhibitory concentration of cyadox against clinical strains of *Clostridium perfringens*

A total of 60 clinical isolates collected from the Guangdong province of China were studied. The MICs of these strains were determined under anaerobic conditions in accordance with the double dilution agar method described by the Clinical and Laboratory Standard Institutes (CLSI; M11-A8). The strains were inoculated onto the supplemented Brucella agar plates using a steer multipoint inoculator to obtain a final concentration of approximately 10^5^ CFU/spot. After inoculation, the plates were incubated under anaerobic conditions (85% N_2_, 10% CO_2_, and 5% H_2_) for 48 h at 37 °C to determine the MICs. The MIC was defined as the lowest concentration that yielded no visible growth or a marked reduction in growth compared to the growth controls. The test strains were cultured in parallel to a control strain under both anaerobic and aerobic conditions. *Bacteroides fragilis* ATCC 25285 served as the quality control strain. The MIC_50_, and MIC_90_ were calculated using SPSS 16.0.

### Determination of minimum inhibitory concentration, minimum bactericidal concentration, mutant prevention concentration, and post-antibiotic effect of the CPFK 122995 isolate

The MIC and MBC for the CPFK 122995 isolate with the highest pathogenicity were determined *in vitro* and *ex vivo* using the microdilution technique. Determination of MBC was performed by inoculating the supplemented Brucella agar plate with 100 μL of suspension from the initial MIC testing with no obvious bacteria. Inoculated plates were inverted and incubated under anaerobic conditions. Viable cells were counted after overnight incubation, and the MBC was determined as the concentration that reduced the viable organism count by ≥3log_10_ over 24 h. The drug carryover effect was reduced by ≥250-fold sample dilution into the agar plate.

The MPC of cyadox was determined using the agar dilution method. For each of the *C*. *perfringens* strains, 10^10^ CFU/mL was inoculated onto the supplemented Brucella agar plates containing serial dilutions of cyadox (1 × MIC, 2 × MIC, 4 × MIC, 8 × MIC, 16 × MIC, and 32 × MIC). The plates were then incubated in an anaerobic chamber at 37 °C, and the MPC was defined as the lowest concentration that yielded no visible bacterial growth after 72 h.

For the PAE determination, logarithmically growing cultures of *C*. *perfringens* at an initial inoculum of 1 × 10^6^ CFU/mL were exposed to a cyadox concentration equivalent to 1-, 2-, and 4-times the MIC for 1 or 2 h. The media containing cyadox was removed by 1000-fold dilution with broth medium, and the continued suppression of bacterial growth was monitored over time. The PAE was defined as the time required for the antimicrobial-treated bacterial to increase in number by 1log_10_ CFU/mL minus the value determined for the non-treated cultures of the same bacteria.

### *In vitro* and *ex vivo* time-kill study

The *in vitro* kill curves of cyadox against *C*. *perfringens* were established by plotting time versus log_10_ CFU/mL. The CPFK122995 strain from a mid-log phase culture was added to 10 mL of Brucella broth to give a starting inoculum of 10^6^ CFU/mL. Cyadox was added to obtain serial concentrations corresponding to 1/4 × MIC, 1/2 × MIC, 1 × MIC, 2 × MIC, 4 × MIC, 8 × MIC, 16 × MIC, and 32 × MIC. The tubes were placed in a 37 °C anaerobic chamber and the bacterial count (CFU/mL) was determined by the agar dilution method for each tube after incubation for 1, 2, 4, 6, 8, 12, and 24 h. Briefly, each culture sample was subjected to 10-fold serial dilutions with sterile saline, then 100 μL of each dilution was spread onto the agar plates. The plates were incubated at 37 °C under anaerobic conditions, and the viable colonies were counted after 24 h. Each concentration was tested in triplicate. The limit of detection was 10 CFU/mL.

The *ex vivo* kill curves were determined as described above using the intestinal fluid samples obtained from pigs at different time points after oral administration. The tubes containing the bacterial cultures and sterile intestinal fluid samples were incubated at 37 °C under anaerobic conditions, and the number of viable organism was determined after 1, 2, 4, 8, 12, and 24 h. Results were expressed as CFU/mL with a detection limit of 10 CFU/mL.

### Pharmacokinetic-pharmacodynamic integration and modeling

To determine the PK/PD integration of cyadox in the ileum fluid, parameters representing the bacteriological outcome including T > MIC (time for which cyadox concentration is above the MIC), maximum concentration (C_max_)/MIC ratio, and AUC over 24 h (AUC_0–24h_)/MIC ratio were calculated using *in vitro* MIC and *in vivo* PK parameters.

For PK/PD modeling, AUC_0–24h_/MIC data obtained from *ex vivo* bacterial kill curves over 24 h were modeled using the inhibitory sigmoid E_max_ model. The model was described by the Hill equation (1):

1 $$E={E}_{\max }-\frac{({E}_{\max }-{E}_{0})\cdot {C}^{N}}{{C}^{N}+E{{C}_{50}}^{N}}$$

where *E* is the antibacterial effect measured as the change in log_10_ CFU/mL in the ileum fluid sample after 24 h incubation compared to the initial inoculum, *E*_*max*_ is the maximum effect of cyadox (log_10_ CFU/mL reduction) after 24 h incubation in the ileum fluid sample compared to the initial inoculum, *E*_*0*_ is the antibacterial effect (change in log_10_ CFU/mL) after 24 h of incubation in a drug-free pig ileum fluid sample, *EC*_*50*_ is the AUC_0–24h_/MIC of cyadox required to produce 50% of the maximum antibacterial effect, *C* is the AUC_0–24h_/MIC of cyadox in the effect compartment (*ex vivo* site), and *N* is the Hill coefficient describing the slope of the AUC_0–24h_/MIC relationship. The PK/PD parameters of the Hill equation were calculated using non-linear regression software (WinNonlin, 5.2; Pharsight Corporation).

Based on the results of PK-PD modeling for the relationship between AUC_0–24h_/MIC and the *ex vivo* antibacterial effect, three levels were quantified to describe the antibacterial effect of cyadox administration, including: (1) bacteriostatic action (no change in bacteria count, E = 0), (2) bactericidal action (99.9% reduction in bacterial count, E = −3), and (3) bacterial elimination (99.99% reduction in bacterial count, E = −4).

### Daily dosage prediction

Assuming pharmacokinetic linearity, the predicted daily doses were calculated by equation^44, 45^ (2):

2 $${\rm{Do}}se=\frac{CL\times {(AUC/MIC)}_{BP}\times MIC}{F\times fu}$$

where *CL* is the clearance per day, *(AUC/MIC)*_*BP*_ is the targeted endpoint for optimal efficacy in hours, the *MIC* is the target pathogen, *F* is the bioavailability factor (from 0 to 1), and *fu* is the free fraction of the drug (from 0 to 1). In this study, the *F* and *fu* = 1. The daily dose was computed by Monte Carlo Simulations using Oracle Crystal Ball software (Oracle Corporation, Redwood Shores, CA, USA) with the following data inputs: (1) the distribution of Cl/F obtained for six individual pigs in the pharmacokinetic study; (2) ileum fluid AUC_0–24h_/MIC ratios obtained from PK-PD modeling; and (3) the distribution of MIC values for the clinical isolates. The probabilities of distribution for daily doses were run for 100,000 trails. The daily dose required to achieve TAR of 50% and 90% for bacteriostatic, bactericidal and bacterial elimination effects were determined.

### Statistical analyses

Data are presented as mean ± SEM or SD. The arithmetic, geometric and harmonic means were determined, as appropriate, for each pharmacokinetic variable. Differences between the ileum fluid and plasma samples were determined by ANOVA using Prism software (Graphpad Software Inc., London, UK).

### Ethics statement

The use of pigs in this study was conducted according to the Animal Experiment Ethical Inspection of Laboratory Animal Center, Huazhong Agricultural University, Wuhan, China (HZAUSW-2016-007). All efforts were made to minimize the suffering of the animals.
